# Efficacy and tolerance of the topical application of potassium hydroxide (10% and 15%) in the treatment of molluscum contagiosum: Randomized clinical trial: Research protocol

**DOI:** 10.1186/1471-2334-11-278

**Published:** 2011-10-19

**Authors:** Josep R Marsal, Ines Cruz, Concepcio Teixido, Olga Diez, Mireia Martinez, Gisela Galindo, Jordi Real, Joan A Schoenenberger, Helena Pera

**Affiliations:** 1 Primary Care Research Institute IDIAP Jordi Gol, Catalan Institute of Health. Rambla de Ferran, 44, 3ª. Lleida, Spain; 2 Ronda Health Center, Catalan Institute of Health. C/de la Mercè, 5. Lleida, Spain; 3 Rambla de Ferran Health Center, Catalan Institute of Health. Rambla de Ferran, 44. Lleida, Spain; 4 University Hospital Arnau de Vilanova. Pharmacy Unit. Institut de Recerca Biomèdica de Lleida. Alcalde Rovira Roure, 80. Lleida. Spain; 5 Primary Care Research Institute IDIAP Jordi Gol, Catalan Institute of Health. Gran Via de les Corts Catalanes, 587, àtic. Barcelona. Spain

## Abstract

**Background:**

Molluscum contagiosum is a non-severe pediatric viral infection. Because it is highly contagious and current treatments have negative aesthetic and psychological effects, we want to test an alternative treatment in the primary care setting, consisting of two different concentrations of potassium hydroxide solution.

**Methods/design:**

The study design is a double-blind, randomized clinical trial, using three types of topical treatment. The treatment consist of daily applications of potassium hydroxide (KOH) in aqueous solution at 10% and 15% concentration, and a placebo administered in the control group. Four follow-up visits (at 15, 30, 45 and 60 days) are planned to evaluate treatment effectiveness and patient tolerance.

The main outcome measure of the trial will be the healing rate, defined as lesion disappearance in the affected zones after the topic application of the experimental treatment. Secondary measures will be the principal characteristics and evolution of the affected zone (surface area, number of lesions, size and density of lesions), treatment tolerance (hyperpigmentation, itching, burning, pain), recurrence rate and the natural evolution of lesions in the control group.

**Discussion:**

KOH can potentially be an effective and safe treatment for MC in primary care, and can also reduce referrals to dermatologists and hospital pediatric departments. In addition, KOH may be a valid and less expensive alternative to current invasive treatments (surgical excision).

**Trial Registration:**

ClinicalTrials.gov: NCT01348386

## Background

Molluscum contagiosum (MC) is a viral infection of the skin [1] produced by a member of the Poxvirus family, the genus Molluscipoxvirus [2]. The symptoms are the appearance of pearly, usually umbilicated papulae. The lesions are completely asymptomatic and can affect any part of the body, usually affecting the thorax or lower extremities. They usually appear asymmetrically in previously injured zones (wounds or atopic dermatitis). Transmission requires direct contact with the infected host or contaminated fomites. MC frequently affects children using community swimming pools or siblings using the same washing sponges or towels [1-5]. The period of incubation usually oscillates between three and twelve weeks. Clinical diagnosis is based on the detection of the characteristic appearance of lesions. Differential diagnosis includes folliculitis, follicular hyperkeratosis, viral verrucas and insect bites. Lesions cause intense feelings of embarrassment not only to parents but also to children, which sometimes result in school absenteeism and curtailed activities. The natural history of MC is spontaneous regression within six to eighteen months. However, because it is highly contagious, new lesions from the incubating virus may appear later. There is considerable debate as to whether it is necessary to treat the infection. The aesthetic or psychotherapeutic effects of treatment need to be evaluated, as well the ease of transmission of the virus.

Treatment consists of different methods to destroy infected tissue together with the virus. These can be: crude/bloody (curettage, electrocoagulation, clamping, etc.), excessive (oral cimetidine, oral isotretinoin), or topical therapies (salicylic acid, tretinoin or potassium hydroxide) [6]. Since 1999, some studies have been published that satisfactorily tested the use of potassium hydroxide (KOH) [2,7-9] in different concentrations, and imiquimod [10]. However, none of these were tested in a standard pediatric primary care clinic.

Four published studies were found in a clinical bibliographic search. They all concluded that KOH solution is an effective and safe treatment for MC. A notable heterogeneity existed among study methodologies, with different KOH concentrations used for treatment and/or studies lacking a control group.

Only one study was designed as a clinical trial, the others were descriptive designs. All the studies were done in the context of medical specialties other than pediatrics.

The present study will be conducted in a primary care setting, as the point of first contact within the National Health Service (NHS). The aim is to assess the efficacy of topical KOH treatment in 10% and 15% aqueous concentrations, versus a control (placebo) group in a pediatric clinic in a primary care center (PCC) in Lleida (Spain).

A randomized clinical trial (RCT) design has been chosen, with the objective of providing the maximum level of evidence. The treatment will be administered to three groups - one control and two treatment groups. This design will compare both treatment groups to the control group as well as compare the efficacy of the differences doses.

We hypothesize that the daily application of KOH treatment for MC is safe and affective in pediatric patients. Our bibliographic research has identified a paper [7] that estimates a 20% rate of successful resolution with placebo treatment and a 70% success rate with KOH treatment, with a mean treatment period of 54 days. Another study [8] without a control group and twice daily applications of 10% KOH reported 32 successful resolutions of 35 cases during a six month period.

This trial has been approved by the Ethical Committee of Clinical Trials of the IDIAP Jordi Gol (Primary Care Research Institute, Spain)

## Methods/design

### Objectives

#### 1.1 Principal objective

Evaluate the efficacy (disappearance of lesions) of treating MC with daily topical applications of KOH (in concentrations of 10% and 15%).

#### 1.2 Secondary objectives

- To evaluate reductions in the number, size and density of MC lesions in affected areas.

- To evaluate the time lapsed between treatment application and cure (disappearance of the lesions).

- To evaluate tolerance to the treatment.

- To describe the natural evolution of the infection with respect to the number, size and density of lesions in affected areas, based on findings in the control group.

- To compare the differences in efficacy of and tolerance to treatment in the three study groups.

### Trial design

#### 1.1 Summary of trial design

The study will be a double blind clinical trial with random treatment allocation. Patients will be randomized into three parallel treatment arms. Treatment will consist of the application of topical 10% or 15% KOH in an aqueous solution for the two treatment groups, compared to a placebo-treated control group.

A total of five appointments are planned, one baseline and four follow-up visits. Screening will be done during the baseline visit to verify that all patients fulfill the inclusion and exclusion criteria, and have signed a written consent form, in addition to recording sociodemographic variables and a clinical history.

The efficacy of and tolerance to the treatment, as well as the appearance of new lesions, concomitant treatments and adverse affects will be evaluated in the follow-up visits.

During the baseline visit, the patient will be diagnosed and essential information will be recorded (inclusion/exclusion criteria, sociodemographic variables and clinical assessment). All valid/evaluable patients will be assigned a randomization code, up to a maximum of 60 patients who fulfill the inclusion/exclusion criteria. Neither the patient, their parents, nor the pediatrician will know to which group the patient has been assigned. Four follow-up visits are planned, at 15, 30, 45 and 60 days (± 3 days) from baseline. Each visit will include a clinical assessment, where the appearance of new lesions will be recorded. The efficacy of treatment and the tolerance of treated lesions since the last visit will be studied. A photograph of the affected area will be taken. If the patient misses the follow-up visit, compliance will be noted in the completion worksheet (which will be filled out in all remaining cases at 60-day follow-up). The randomization code will be revealed if the attending physician believes this to be appropriate. In this case the patient will be removed from the study, filling out the completion worksheet.

#### 1.2 Description of the measures taken to minimize/avoid bias

##### 1.2.1 Randomization and codebreaking

Randomization will be centralized at the Pharmacy Department of the hospital of reference, the Arnau de Vilanova University Hospital (HUAV).

Patients will be randomly allocated to one of the study groups. Randomization will increase the probability of distributing patients to the three groups homogenously.

##### 1.2.2 Blinding

The vials of solution to be studied will be prepared as follow:

- KOH 10%: 10 grams of KOH dissolved in purified water, 100 ml

- KOH 15%: 15 grams of KOH dissolved in purified water, 100 ml

- Placebo: 100 milliliters of saline solution

The vials will be indistinguishable, regardless of content, and will be stored at room temperature. A mask and gloves are required for preparing the solutions.

##### 1.2.3 Other measures taken to minimize/avoid bias

HOMOGENEOUS APPLICATION: Each patient will receive a bottle of the solution assigned (identical bottles), depending on their randomization code. The parents/guardians of the patient will be shown how to apply the solution to the lesions, and written instructions will be provided, as follows: the treatment should be applied once per day with a cotton swab soaked in the solution, unless the lesion shows signs of INFLAMMATION OR IRRITATION, at which point the TREATMENT SHOULD BE STOPPED (parents should write down the date in order to inform the physician at the next follow-up visit).

#### 1.3 Primary and secondary endpoints/outcome measures

Efficacy will be assessed in each of the areas detected in the previous visit. If there are new areas affected, they will be evaluated at the next visit (a description of the new lesion will be recorded in the topographic section of the patients record).

The principle efficacy variable (primary endpoint) will be calculated for each of the affected areas, as an indicator variable: (1. There is a lesion in the area/0. No lesion in the area) at 60 days from baseline. Each patient will be considered cured if they have no lesions in any of the treated areas.

Secondary efficacy variables (secondary endpoints) will be calculated for each location as follows:

- Number of lesions.

- Extent of area affected.

- Mean size of lesions.

○ < 0.5 cm.

○ 0.5 cm. - 1 cm.

○ > 1 cm.

- Efficacy of method: Ordinal scale with five categories.

○ Worsening (net increase in number of lesions or growth of existing ones).

○ No changes *(no modifications to the lesion).*

○ Slight improvement *(disappearance of up to 50% of lesions).*

○ Moderate improvement *(disappearance of more than 50% of lesions).*

○ Cure (*complete disappearance of lesions*) (primary endpoint).

- Digital photograph.

- Lesion density*: Number of lesions/Extent of affected area

*NOTE: If the parents locate new lesions between visits they should NOT treat them until the physician has clinically evaluated their topography*.

#### 1.4. Study population

The reference population will be pediatric patients treated in primary care with a clinical diagnosis of MC.

##### 1.4.1. Inclusion criteria

- Who are diagnosed with a MC infection (clinical diagnosis).

- Who are between the ages of two and six.

- Whose parents or guardians have provided written informed consent for participation.

##### 1.4.2. Exclusion criteria

- Immunocompromised patient (congenital or acquired).

- Patient has received other topical treatment within the last month.

- Patient has lesions on face, neck or genital area.

- Patient who, in the view of the attending physician, will not comply with treatment and/or scheduled visits.

#### 1.5 Informed consent

In accordance with Spanish law, parents/guardians must personally sign and date the latest approved version of the informed consent form before any study specific procedures are performed.

Written and verbal versions of the Participant Information Sheet and the Informed Consent Form will be presented to the participants (parents/guardians), detailing no less than: the exact nature of the study; the implications and constraints of the protocol; the known side effects and any risks involved in taking part. It will be clearly stated that the participant is free to withdraw from the study at any time for any reason without prejudice to future care, and with no obligation to provide the reason for withdrawal.

The participant will be allowed as much time as they wish to consider the information, and the opportunity to question the Investigator, their physician or other independent parties to decide whether they will participate in the study. Written informed consent will then be obtained by means of the participant's dated signature and the dated signature of the person who presented and obtained the informed consent. The person who obtained the consent must be suitably qualified and experienced, and have been authorized to do so by the Principal Investigator. A copy of the signed ICF will be given to the participants. The original signed form will be retained at the study site.

#### 1.6 Screening and eligibility assessment

All parents/guardians of patients diagnosed with MC will be provided the necessary written information for study enrollment (information sheet) as well as clarification of any questions they may have. Once the patient has been determined to be valid (fulfillment of inclusion/exclusion criteria and written informed consent), they will be assigned a randomization code. The baseline visit will be done and then the patient will be scheduled for the next visit. Each parent/guardian will receive precise instructions for how to apply the treatment and will receive a vial of their designated treatment solution coded with the patient's number. The patient will be scheduled for the four follow-up visits at 15, 30, 45 and 60 days from the first treatment application.

#### 1.7 End of trial assessment

Each study participant can leave the study for two reasons:

1) Loss to follow-up (before the end of follow-up protocol, without successful treatment).

a. Lack of efficacy.

b. Choice of the patient.

c. Adverse effects.

d. Loss to follow-up (missing scheduled visits).

e. Breaking the blind.

f. Other.

2) Completion of follow-up.

g. Attended all scheduled visits

h. Success of treatment

A completion form should be filled out for all patients, whether they have finished the study or not.

#### 1.8. Statistical methods and sample size

##### 1.8.1. Statistical analysis

The statistical analysis will be carried out on several different samples of the study population. The valid or valuable patient group is defined as those patients that fulfill all the inclusion criteria, none of the exclusion criteria, whose parents have provided consent for their participation and who have been assigned a treatment (randomized code). The safety sample is defined as the group of valid patients who have received treatment in the study at least once, whether or not its effectiveness has been assessed (who came to at least one follow-up visit). The intention to treat (ITT) sample is defined as the group of valid patients who have received treatment at least once and who have been assessed for its effectiveness at least once. The per protocol (PP) sample is defined as the group of patients that make up the ITT sample, have been assessed for efficacy and have not violated the protocol in any of the following ways:

- Violation of blinding.

- Greater than 10% missed treatments.

- Loss to follow-up by missing a scheduled visit.

The unit of analysis is the patient. Nevertheless, each patient can present multiple affected areas. An area is considered to be a set of lesions whose perimeter does not coincide with another affected area, with a single area being possible. No more than 15 affected areas per patient are expected.

A descriptive analysis of all the variables collected will be done, expressing quantitative variables as means, medians, minimums, maximums and standard deviations (SD), and categorical variables as frequencies and percentages.

The homogeneity of the study groups with respect to the following baseline parameters will be compared:

Demographic variables

Initial topography

Clinical history of associated diseases

History of treatments received (> 1 month)

To determine the existence of an association between a group and secondary efficacy variables at each period of follow-up, the single-factor ANOVA test will be used for quantitative variables (extent, number of lesions, etc.) and the chi-squared test will be used for categorical variables (cure).

In the case that the ANOVA cannot be used because a quantitative parameter does not fulfill the application criteria (non-normality/heteroscedasticity) the data will be transformed (logarithmic) from the original parameter, or a non-parametric analog test (Kruskal-Wallis) will be used. In all 2 × 2 comparisons (Group I, II vs. Placebo), the significance level will be adjusted for multiple comparisons.

The evolution of the quantitative efficacy parameters will also be assessed, by group, for the different time periods, using a repeated measures analysis.

The relationship between the baseline variables and the efficacy variables will be assessed at the end of the study, and in the case that an association is found, a multivariate analysis will be carried out to assess possible confounding factors of the group effect on adjusted efficacy: a multiple regression analysis will be used in the case of quantitative efficacy variables and a multinomial regression will be used in the case of qualitative variables.

The time from treatment to cure will be calculated as the difference between the date of first treatment and the disappearance of lesions or the end of the study (treatment initiation). Survival tables and the Kaplan-Meier statistic will be calculated to compare cure times between the different treatment groups. In the case that associations are found between efficacy variables and baseline conditions, a Cox proportional risk model will be adjusted.

Data analysis will be by ITT, by protocol and by safety sample.

In all cases a p-value below 0.05 will be considered significant.

Data will be analyzed using the statistical package "R": http://www.r-project.org.

##### 1.8.2. Sample size calculation

The calculation of the sample size takes as its primary outcome treatment efficacy - the rate of complete elimination of MC at the final follow-up visit (60 days), as specified in a published clinical trial [7].

Based on this clinical trial, we expect a 20% cure rate in the control group and a 70% cure rate in one of the experimental treatment groups. To be able to detect these differences with a chi-squared test of two independent samples with a power of 80% at the 5% significance level, and with an estimated follow-up loss rate of 20%, there must be 20 patients per group.

#### 1.9. Quality control and quality assurance

In order to ensure the Quality of the data the delegated organization will:

• Perform regular monitoring according to ICH GCP guidelines. Data will be evaluated for compliance with the protocol and accuracy in relation to source documents. Monitors will verify that the clinical trial is conducted and data are generated, documented and reported in compliance with the protocol and the applicable and local regulatory requirements.

• Provide instructions and training to the sites involved in the trial.

• Review the CRF data.

• Detail of any other steps taken to ensure research quality.

## Discussion

Currently, there is no satisfactory treatment for the rapid and non-invasive treatment of MC lesions, so pediatricians normally allow the infection to run its course, with duration of between six months and a year. This situation imposes considerable limitations on the patient's lifestyle, especially regarding the use of public facilities (gyms, pools) due to the elevated infectivity of the virus, and psychological and social effects especially in children.

KOH may be a good therapeutic alternative if it is effective, easy to apply, can be self-administered, has minimal side effects and is inexpensive. The few published studies on the subject do not provide definitive evidence about KOH as a treatment for MC, given that these studies have not compared different solution concentrations and placebo.

The results of our clinical trial will make it possible to definitively assess all these aspects of this treatment. Furthermore, conducting the study in a pediatric primary care setting, the gateway to the Spanish national health system, makes it possible to evaluate the utility of the treatment at the primary care level, addressing the majority of cases that could benefit from this treatment while also decreasing referrals to hospital specialists.

## Competing interests

The authors declare that they have no competing interests.

## Authors' contributions

CT and OD conceived the study, and conducted the recruiting and intervention. IC and GG participated in the design and coordination of the study. IC drafted the manuscript. JRM and JR participated in the design of the study and performed the statistical analysis. MM and JS participated in the study design, prepared, bottled and blinded the solution and contributed to follow up. HP participated in the study coordination. All authors read and approved the final manuscript.

## Pre-publication history

The pre-publication history for this paper can be accessed here:

http://www.biomedcentral.com/1471-2334/11/278/prepub
